# Small interfering RNA (siRNA)-based therapeutic applications against viruses: principles, potential, and challenges

**DOI:** 10.1186/s12929-023-00981-9

**Published:** 2023-10-16

**Authors:** Hara Kang, Yun Ji Ga, Soo Hyun Kim, Young Hoon Cho, Jung Won Kim, Chaeyeon Kim, Jung-Yong Yeh

**Affiliations:** 1 Department of Life Sciences, College of Life Sciences and Bioengineering, Incheon National University, Academy-Ro 119, Yeonsu-Gu, Incheon, 22012 South Korea; 2 Research Institute for New Drug Development, Incheon National University, Academy-Ro 119, Yeonsu-Gu, Incheon, 22012 South Korea; 3 Convergence Research Center for Insect Vectors, Incheon National University, Academy-Ro 119, Yeonsu-Gu, Incheon, 22012 South Korea; 4 KU Center for Animal Blood Medical Science, College of Veterinary Medicine, Konkuk University, 120 Neungdong-Ro, Gwangjin-Gu, Seoul, 05029 South Korea

**Keywords:** Antiviral, RNA interference, Small interfering RNA, Therapeutic, Virus

## Abstract

RNA has emerged as a revolutionary and important tool in the battle against emerging infectious diseases, with roles extending beyond its applications in vaccines, in which it is used in the response to the COVID-19 pandemic. Since their development in the 1990s, RNA interference (RNAi) therapeutics have demonstrated potential in reducing the expression of disease-associated genes. Nucleic acid‐based therapeutics, including RNAi therapies, that degrade viral genomes and rapidly adapt to viral mutations, have emerged as alternative treatments. RNAi is a robust technique frequently employed to selectively suppress gene expression in a sequence-specific manner. The swift adaptability of nucleic acid‐based therapeutics such as RNAi therapies endows them with a significant advantage over other antiviral medications. For example, small interfering RNAs (siRNAs) are produced on the basis of sequence complementarity to target and degrade viral RNA, a novel approach to combat viral infections. The precision of siRNAs in targeting and degrading viral RNA has led to the development of siRNA-based treatments for diverse diseases. However, despite the promising therapeutic benefits of siRNAs, several problems, including impaired long-term protein expression, siRNA instability, off-target effects, immunological responses, and drug resistance, have been considerable obstacles to the use of siRNA-based antiviral therapies. This review provides an encompassing summary of the siRNA-based therapeutic approaches against viruses while also addressing the obstacles that need to be overcome for their effective application. Furthermore, we present potential solutions to mitigate major challenges.

## Background

The coronavirus disease 2019 (COVID‐19) pandemic caused by SARS-CoV-2 has underscored the imperative for preparedness with intervention technologies against global outbreaks. mRNA-based vaccines such as Comirnaty^®^ and Spikevax^®^ have been successfully developed and applied against COVID-19 [1, 2], demonstrating the potential of mRNAs in fighting emerging infectious diseases. In addition to mRNA-based vaccines, the emergence of nucleic acid-based therapeutics capable of degrading viral genomes and adapting to mutations are alternative treatments [3, 4].

RNA interference (RNAi) is a powerful strategy for targeting and suppressing genes [5, 6] and has been applied to gene function exploration [7–10] and antiviral therapies. Nucleic acid-based therapeutics, such as RNAi, show more adaptability in than traditional antiviral drugs, making them suitable to combat emerging infectious diseases. RNAi is mediated by small double-stranded fragments known as small interfering RNAs (siRNAs), which induce gene silencing via posttranscriptional regulation [5, 11]. The precision of siRNAs in targeting viral RNA has led to siRNA-based treatments for various viral diseases, including COVID-19 [12].

## General overview of antiviral RNAi

**Fig. 1:**
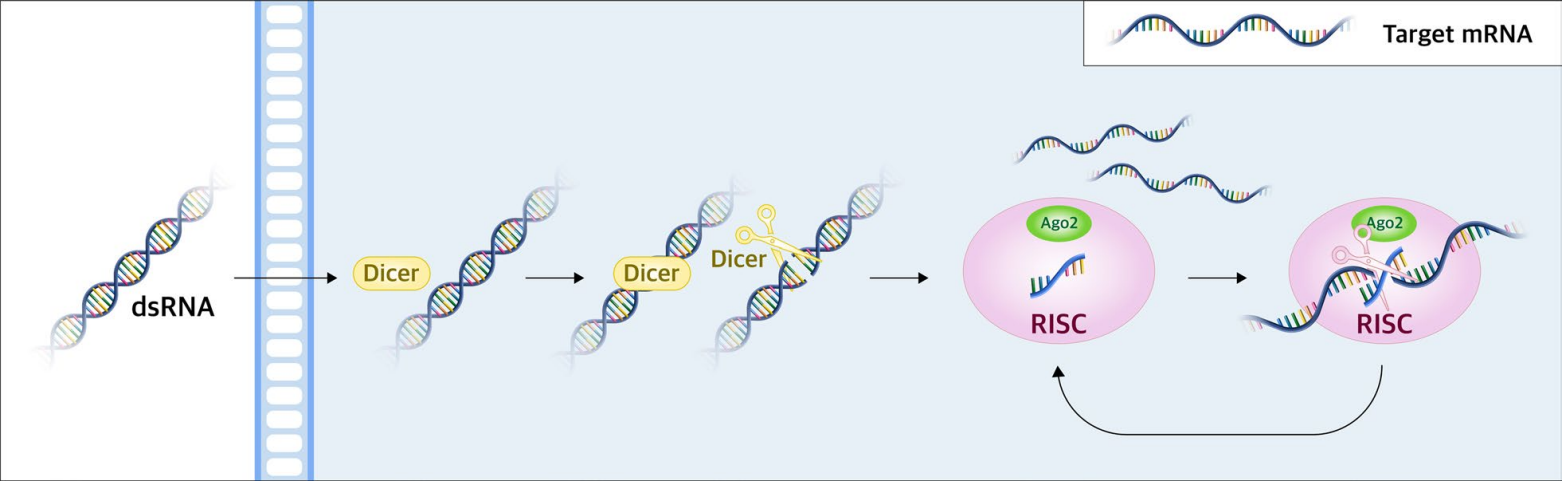
The mechanism of RNA interference (RNAi). Extended double-stranded RNA (dsRNA) can be delivered to the cytoplasm, where it undergoes cleavage to generate small interfering RNA (siRNA) by the enzyme Dicer. siRNAs can also be introduced directly into a cell as long dsRNAs and then cleaved by RNase III (Dicer) in the cytoplasm to become small dsRNAs. dsRNA is processed into 21–30 nt-long short siRNA molecules that act as modules in the silencing mechanism [214]. Subsequently, siRNA is integrated into a multiprotein complex known as the RNA-induced silencing complex (RISC), leading to the fragmentation of the RNA sense strand through the action of Argonaute 2 (Ago2). The activated RISC-siRNA complex actively searches for, attaches to, and degrades mRNA molecules that have complementary sequences, thereby causing suppressing target gene expression. Moreover, the activated RISC-siRNA complex can be reactivated to target and eliminate additional mRNA molecules with identical sequences [72, 215, 216]

RNAi is a conserved biological process induced by noncoding RNAs that inhibits gene expression by blocking the transcription or translation of specific genes [5, 13–16]. RNAi therapeutics have had a clear impact in reducing the expression of disease-associated genes since their development in the 1990s [17]. The activation of RNAi using synthetic siRNA or short hairpin RNA (shRNA) is a common strategy of gene knockdown in mammalian cells. Through antiviral RNAi programs dsRNA replicative intermediates are identified and processed into 21–23 nucleotide siRNAs with perfectly paired bases by Dicer [18]. These antiviral siRNAs guide Argonaute proteins within the RNA-induced silencing complex (RISC) to cleave cognate viral RNAs. Synthetic siRNAs are also employed, bypassing Dicer (Fig. 1). Theoretically, an appropriately designed siRNA can be used to silence almost any viral gene. Thus an RNAi strategy covers a wider antiviral therapeutic range than conventional small-molecule drugs.

The RNAi-based genetic regulatory mechanism is observed in various organisms, such as plants, animals, and fungi and is based on small RNA triggers that fine-tune gene expression [11, 14]. Among extensively studied noncoding RNAs, the most prominent are both exogenous and double-stranded siRNAs and endogenous and single-stranded microRNAs (miRNAs). These RNAs play crucial roles in gene regulation [19]. Derived either from exogenous double-stranded RNA sequences or through miRNA-directed gene silencing, siRNAs and miRNAs are integral components of gene control machinery. miRNAs are transcribed from cellular genomes and regulate the expression of endogenous genes [20–22]. In contrast to miRNAs, which can influence multiple mRNA targets through partial complementarity, siRNAs exhibit the capacity to precisely target and cleave a specific mRNA [23–25]. Therefore, each siRNA can be meticulously engineered to target a particular gene, restricting its inhibitory action solely against the designated gene [26]. This review focuses only on exogenous siRNAs and describes the challenges to overcome and advancements made with antiviral RNAi therapeutics.

## siRNA applications as potential therapeutics to combat viral infections

Therapies based on siRNAs are highly promising and adaptable treatments for viral infections. RISC-mediated RNA cleavage is restricted to cytoplasmic target molecules, and since all viruses hijack cellular translation machinery to express their own proteins, targeting virally encoded cytoplasmic mRNAs is a common strategy, at least in theory, to mitigate infection with viruses susceptible to inhibition [27]. Exogenous siRNAs, designed to mediate the degradation of viral RNA targets, are typically introduced into cells with synthetic or vector-based delivery systems [28].

siRNA therapies are flexible approaches to viral infection treatment. RISC-mediates cleavage of virally encoded cytoplasmic mRNAs to inhibit virus replication. Exogenous siRNAs degrade viral RNAs via RNAi pathways [28]. As soon as the genome of a novel virus is identified, RNAi is a reliable infection-mitigating strategy. siRNA-based RNAi therapies address the cause of infection, not merely palliate the symptoms of the disease in both prophylactic and curative settings. In summary, siRNA-based antiviral therapy efficiently controls infectious diseases by mediating posttranscriptional gene silencing [29].

### siRNA are alternative therapeutic platforms to fight viral infections

For siRNA-based therapeutic applications against viral infection, two categories of siRNAs can be exploited. The first type of siRNAs target viral proteins necessary for viral growth and replication, and the other type consist of host factors responsible for the intracellular entry and trafficking of viruses [3]. This review focuses mainly on the knowledge of siRNA-based therapeutic applications involving viral targets.

siRNAs are modular short viral nucleic acid segments that do not interfere with the human genome [30–33]. For example, most of small-molecule drugs used for human immunodeficiency virus type 1 (HIV-1) therapy are directed to either reverse transcriptase or protease [34]. Rather than targeting functional domains of viral proteins, some siRNAs target a short viral nucleic acid segments, making even a small viral genome a rich source of potential targets.

The advancements made in siRNA therapeutics have generally been realized in a series of stages. Primarily, siRNAs are precisely designed and synthesized to identify the most consequential sequences in the viral mRNA strand. This process involves a combination of experimental and computational techniques. Following the confirmation of their potency and selectivity through in vitro assessments, antiviral siRNA strands can be further stabilized via the optimization of chemically modified nucleotides. Finally, multiple delivery strategies are available because of the recent development of various formulations and methods.

### Mechanisms underlying antiviral siRNA functions

The mechanism underlying siRNA antiviral treatments involves targeting and initiating transcription termination of most-critical mRNAs that encode essential viral proteins. The ability of siRNAs to silence virtually any gene means that viral genes essential for replication can be targeted [35] to inhibit viral replication and spread [36]. The ability of RNAi machinery to adjust rapidly to viral mutations and its potential for simultaneous targeting of multiple viral genomic sites contribute to the attractiveness of this strategy. Notably, after employing computational methods to prevent viral escape, siRNA therapies can be used for quickly addressing emerging viral infections. Efficient gene design and delivery technologies have improved siRNA-based therapeutic effectiveness.

### Clinical perspective on antiviral siRNA

#### Respiratory syncytial virus (RSV)

At present, there are no authorized antiviral therapies based on RNAi. The first RNAi therapeutic to be entered into human clinical trials was ALN-RSV01, developed by Alnylam Pharmaceuticals [37]. ALN-RSV01 consists of a sole siRNA designed to target the mRNA of the nucleocapsid protein of the respiratory syncytial virus (RSV) [38, 39] and was explored for the potential treatment or prevention of RSV infection.

ALN-RSV01 was entered into a phase IIb clinical trial with RSV-infected lung transplant patients, and a naked siRNA was administered via the respiratory route [40]. However, the trial did not meet efficacy targets by the endpoint, and marginally decreased the incidence or progression of bronchiolitis obliterans syndrome in the patients was not statistically significant. These trials indicated the necessity for further optimization of siRNA stability, potentially involving chemically modified siRNA and nanocarriers to enhance delivery [41].

#### Hepatitis B virus

The antiviral outcomes of VIR-2218, a GalNAc-conjugated siRNA developed by Alnylam Pharmaceuticals, in a phase II study involving participants with chronic hepatitis B virus infection demonstrated that a single siRNA target within the X coding region led to a reduction in hepatitis B surface antigen in both hepatitis B e antigen-negative and hepatitis B e antigen-positive participant populations [42].

JNJ-3989, a siRNA conjugated Gal/NAc that is capable of inhibiting all hepatitis B virus transcripts, exhibited efficacy in a phase II study. It led to a reduction in hepatitis B surface antigen levels in both hepatitis B e antigen-positive and B e antigen-negative patients, and it was well tolerated [43]. In a phase IIb clinical trial [NCT03365947], which enrolled patients with chronic hepatitis B, the effectiveness of a combination of JNJ-3989 with nucleos(t)ide analogs, with or without the capsid assembly modulator JNJ-56136379 (also known as bersacapavir), was investigated. The trial demonstrated that the administration of JNJ-3989 along with nucleos(t)ide analogs with or without JNJ-6379 was well tolerated. Additionally, this treatment led to a sustained decrease in hepatitis B surface antigen levels for up to 336 days after the last dose with JNJ-3989 was administered [44].

In a recent phase IIb trial [NCT03982186] established to evaluate JNJ-3989 in combination with nucleos(t)ide analogs, it was observed that although treatment with JNJ-3989 resulted in a dose-dependent response, meeting the nucleos(t)ide analog-mitigating criteria, it rarely led to complete clearance of hepatitis B surface antigen [45].

#### Ebola virus

No Food and Drug Administration-approved Ebola drugs or vaccines are available, with supportive care being the mainstay of therapy. Researchers have investigated siRNAs targeting vital proteins involved in the transcription/translation processes, including viral VP24, VP35, and polymerase L [46–48]. Dunning and colleagues demonstrated that the administration of TKM-130803, a therapeutic comprising two siRNAs (siLpol-2 and siVP35-2) against Ebola virus administered through intravenous infusion to adult patients with severe Ebola virus disease did not lead to increased survival compared to historic controls [49]. Furthermore, Scott et al. found that viral loads were not significantly different at the onset of treatment with TKM-130803 or during treatment (p = 0.1) in subjects who survived or died [50].

#### HIV

Designing RNAi strategies for combating HIV involved greater complexity due to the well-documented challenges. The difficulty in treating HIV-1 infection is attributed to the rapid virus replication rate and its propensity to swiftly escape or the emergence of resistant mutants in response to antiretroviral therapy [27].Furthermore, there are numerous HIV-1 subtypes worldwide, with each type characterized by distinct sequence variations, and the capacity of the virus subtypes to (re)combine, resulting in the circulation of recombinant forms.

The US Food and Drug Administration approved a phase I clinical trial in May 2007 to evaluate RNAi as a nucleic acid therapy using a lentiviral vector capable of infecting nondividing cells for HIV-1 infection [51]. However, siRNAs targeting HIV are still in clinical phase I trials, primarily due to the absence of a comprehensive animal model, which complicates the evaluation of therapeutic agents such as RNAi in vivo [51].

#### Why have we not seen approved antiviral siRNAs two decades after their discovery?

**Fig. 2:**
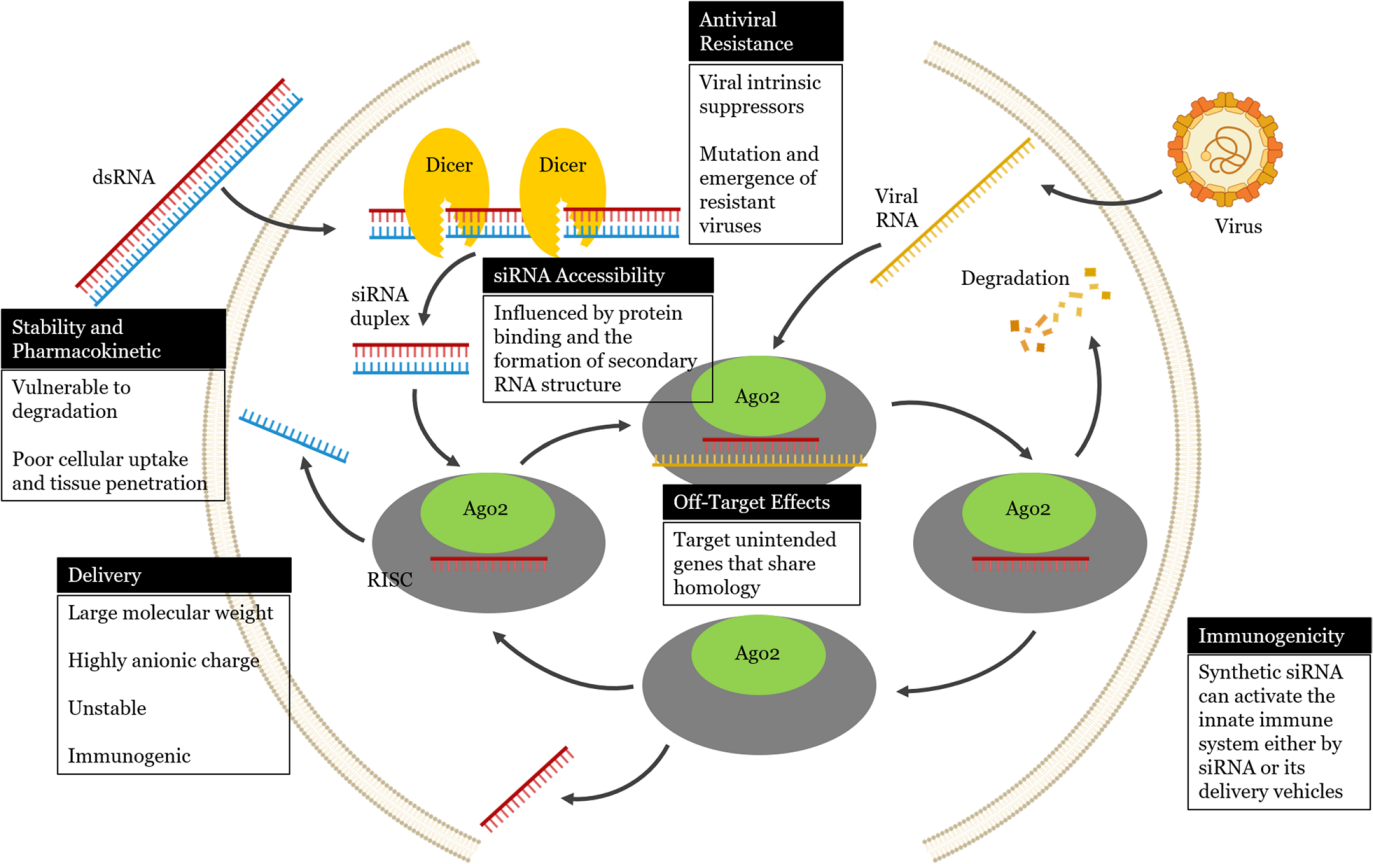
Schematic illustrations showing challenges to small interfering RNA (siRNA) applications against viral infections

The practical application of siRNA for therapeutic purposes holds significant potential and is supported by its proven effectiveness for gene silencing precision, as demonstrated in specific in vitro and in vivo studies. Nevertheless, why have we not yet witnessed the approval of antiviral siRNA two decades after its discovery? The answer lies in the numerous barriers (Fig. 2), discussed in subsequent sections of this review, that must be overcome to fully unlock the potential of this technology [52].

## Challenges of siRNA treatments for viral infections

Theoretically, as previously mentioned, siRNA modalities can be designed and targeted to silence any viral gene of interest, which makes them efficient tools for a broad range of antiviral applications [53]. Despite the promising therapeutic benefits of siRNAs, several obstacles, such as the instability of siRNAs, delivery problems, off-target effects, immunological responses, and development of drug resistance, have hindered their application (Table 1).

**Table 1:** Challenges of small interfering RNA (siRNA) applications to viral infections and strategies to address them

Obstacle	Features	Strategy to address the challenge
Stability and Pharmacokinetics	siRNAs are vulnerable to degradation by nucleases and may show poor cellular uptake and tissue penetration, which can limit their therapeutic effectiveness	· Various siRNA delivery systems include nanocarriers, self-replicating RNA virus vectors, and viral vectors · Modifications of siRNA molecules improve the stability and increase the half-life of siRNA
Delivery	· siRNAs have a large molecular weight of approximately 13 kDa and a highly anionic charge, which makes it difficult for them to cross cell membranes · Unmodified siRNAs are not stable and can cause immune responses	· Use of plasmid and viral vectors as expression cassettes · Encapsulation in synthetic vehicles such as cationic liposomes or nanoparticles and conjugation with cell-penetrating peptides or specific antibodies that target infected cells · Increased efficiency of antiviral siRNA delivery may involve (1) the use of new targeting ligands and chemical probes that specifically bind to surface markers on infected cell populations, (2) increasing the efficacy of siRNA uptake into the cytoplasm, (3) developing materials with low toxicity to widen the antiviral therapeutic window, 4) designing materials with defined degradation products that can be metabolized for repeated dosing, (5) simplifying the formulation procedure, and (6) exploring delivery to organs other than the liver
Off-target effects	Synthetic (exogenous) siRNAs can potentially target unintended genes that share homology with the target viral gene, leading to off-target effects	· Use of siRNAs with high specificity and careful design of the siRNA sequences (analyses of homology and specificity during the design of siRNAs) · Use of multiple siRNA duplexes to increase the certainty of observing the desired phenotype and expression pattern · Identification of the lowest concentration at which antiviral siRNAs are capable of inducing effective gene silencing while preventing off-target effects · Local drug delivery strategies to prevent the off-target accumulation of siRNAs
Immunogenicity	Synthetic siRNA can activate the innate immune system either by siRNA or its delivery vehicles	Proper siRNA design considerations as follows: · Chemical modifications to the RNA backbone (ribose modifications) · Choice of siRNA target sequence (avoidance of sequences prone to inducing inflammatory responses) · Delivery methods (e.g., methods to prevent contamination by remnants of lipid nanoparticle delivery systems) · Use of delivery modalities such as carriers that enhance the cellular internalization · A single siRNA molecule binding to and regulating multiple mRNA copies · High purity of RNA therapeutics reduce the chance of unwanted immune reactions
Antiviral Resistance	· Intrinsic suppressors of viral RNA silencing · Mutation and emergence of viruses resistant to siRNAs	· RNA silencing suppressors do not affect RNAi induced by artificial (synthetic) siRNAs · Combinations of multiple siRNAs targeting separate regions of the viral genome · siRNAs that target highly conserved regions among various isolates
siRNA accessibility	RNAi efficiency is affected by the accessibility of the target RNA, which may be influenced by protein binding and the secondary RNA structure	Targets within the structured viral genome that are highly accessible should be considered

There are various factors that impact the effectiveness of siRNA, such as the guanine‐cytosine (GC) content, nucleotides at siRNA termini, thermodynamic properties, siRNA structure, and accessibility of a target site [54, 55]. To prevent treatment resistance or viral escape due to pathogen mutations, siRNAs need to be developed against conserved sites [56]. It is also recommended to use multiple several site for siRNA targeting to ensure durable viral gene silencing [57]. Additionally, chemically modified nucleotides can increase the stability and reduce the off-target effects of siRNAs [58–60]. For instance, compared to unmodified siRNAs, modified siRNAs used against hepatitis B virus infection exhibited increased half-lives and activity in human serum [61].

### Stability and pharmacokinetics

One of the aforementioned primary challenges, the maintenance of siRNA stability is a significant obstacle to desired therapeutic efficacy of siRNA treatments.

#### Vulnerability of siRNAs to degradation

The foremost extracellular hurdles associated with antiviral siRNA therapy include rapid systemic clearance, rendering siRNAs susceptible to nucleases-mediated degradation. Unmodified RNA molecules are prone to degradation by extracellular nucleases, and their negatively charged nature hampers their ability to traverse the hydrophobic cytoplasmic membrane, negatively affecting both their stability and pharmacokinetics [62–64]. These limitations can hinder cellular uptake and tissue penetration of siRNAs, ultimately compromising their therapeutic effectiveness. Furthermore, the RNA backbone contains ribose, which is highly susceptible to hydrolysis by serum nucleases that cleave phosphodiester bonds [65, 66]. This degradation process prevents the accumulation of intact therapeutic siRNA in targeted tissue after systemic administration [39].

#### Challenges to cellular membrane penetration and endosomal entrapment

Due to their negative charge, naked or otherwise unmodified siRNAs cannot penetrate the cell membrane, which consists of a negatively charged bilayer of phospholipids and functional proteins [67]. Moreover, siRNAs that accumulate in the extracellular environment because they are not taken up by cells are susceptible to rapid degradation by RNases that attack due phosphodiester bonds and phosphatases.

Once internalized, siRNAs must then escape from endosomal compartments and reach the RNAi machinery. Endosomal trapping is another significant obstacle to the success of RNAi-based therapy and has been extensively covered in several excellent reviews [68–71]. Cellular uptake of exogenous siRNA introduced to cells is mediated by encapsulation into endosomes and the subsequent release of the siRNA into the cytoplasm following its endosomal escape [72]. In brief, the siRNA delivery system enters the cells via endocytosis, involving the formation of endocytotic vesicles (endosomes), which is subsequently acidified by the ATPase proton pump in the endosomal membrane. Then, they can be localized to lysosomes, which are also acidified organelles, causing siRNA degradation. The delivery strategy therefore most be designed to prevent the acidification and degradation of siRNA after it enters the cell in an endocytotic vesicle [35].

#### Strategies to increase siRNA stability and protect it from degradation

A way to enhance siRNA stability is to use different types of 2′ sugar modifications; for example, fluorine substitutions, which can increase resistance to endonucleases, can be incorporated into the delivery system [73]. Modifications can also be made to the sugar–phosphate backbone of the siRNA; for example, 2′-fluoro and 4′-thio groups can be added, locked nucleic acids can be incorporated, and phosphorothioation and methyl phosphonation can be induced to increase the stability and half-life of siRNA in serum [74, 75]. Kalke et al. demonstrated high antiviral potency and efficacy of 2′-fluoro-modified antiviral siRNA swarms against herpes simplex virus 1 in human corneal epithelial cells [76]. They also showed that the antiviral effect of the 2′-fluoro-modified swarm was more pronounced than that of the unmodified antiviral siRNA swarm. Dowler et al. revealed that incorporating 2′-deoxy-2′-fluoro-beta-d-arabinonucleotide units into siRNA duplexes increased siRNA activity and substantially increased its stability in serum-containing environments [77]. Another common approach to prevent enzymatic degradation of siRNA is the addition of phosphorothioate backbone linkages at the 3′-end of RNA strands to decrease its susceptibility to exonucleases [78]. In addition, Egli et al. demonstrated that glycol nucleic acid nucleotide or dinucleotide incorporation into an oligonucleotide increased resistance against 3′-exonuclease-mediated degradation [79].

### Delivery challenges

#### Delivery hurdles due to the pharmacological properties of siRNA

The delivery of antiviral siRNA is a significant challenge to siRNA therapeutics development for human use, largely due to the pharmacological properties of siRNA [78, 80, 81]. Compared to small-molecule drugs, siRNAs have a relatively large molecular weight of approximately 13 kDa and carry a high anionic charge resulting from the abundance of phosphate groups, which can range from 38 to 50 groups. These attributes make it difficult for them to cross cell membranes. Moreover, unmodified siRNAs are not stable in the bloodstream and can cause immune responses through their interactions with Toll-like receptors (TLRs) [82]. Upon intravenous administration, siRNAs must pass through the vascular endothelial barrier, diffuse through the extracellular matrix, avoid filtration by the kidneys, and evade uptake by nontargeted cells. Throughout this journey, siRNAs must resist nuclease degradation to retain functionality [78].

Using siRNA delivery vehicles to protect against degradation in the circulatory system is critical for practical siRNA-mediated silencing [66]. Nanocarriers can be designed to shield siRNA from ribonucleases, ensuring stability and resistance to enzymatic degradation. Self-replicating RNA virus vectors and viral vectors are potential attractive alternatives to nanocarriers [83] and are among various siRNA delivery systems that have been employed to enhance endosomal escape [70]. However, it is essential to acknowledge the potential for residual contamination from lipid nanoparticle-based siRNA delivery systems. The lingering presence of siRNA carriers has the potential to profoundly intensify unintended immune responses. Unintended accumulation of nanoparticles can potentially induce both local and systemic inflammatory and immunogenic reactions, possibly leading to the production of autoreactive antibodies [84].

Consideration should also be given to direct injection of antiviral siRNA into infected tissues for cell-specific targeting [85]. To mitigate systemically administered siRNAs that can accumulate in off-target tissues [72], local drug delivery strategies might be used [86, 87].

#### Delivery systems to enhance antiviral siRNA efficacy

Delivery systems for antiviral siRNAs can be classified into vectors and nanoparticles [4, 88]. Plasmid and viral vectors are expression cassettes used to achieve sustained silencing effects. While plasmids are less efficient in delivery compared to viral vectors [89], adenovirus, adeno-associated virus, and lentivirus are commonly used for in vitro and in vivo siRNA delivery [90–94]. Other delivery methods include encapsulation in synthetic vehicles such as cationic liposomes or nanoparticles and conjugation with cell-penetrating peptides or antibodies targeting infected cells [85].

Both lipid- and polymer-based systems share key chemical properties for successful delivery. Polymer-based siRNA delivery vehicles offer structural flexibility and nuclease protection [95]. Cationic materials stabilize interactions and have well-defined polymer morphology, enabling cross-linking, and show promise for efficient siRNA delivery. Cationic lipids in liposomes can overcome the negative charges of siRNAs [28]. Stable nucleic acid lipid particles enhance siRNA stability and transport efficiency [96]. Effective siRNA delivery against hepatitis B virus was demonstrated in mice using stable nucleic acid lipid particles [97]. Moreover, successful delivery systems incorporate cationic/ionizable groups, functional linkers, and lipid tails. Direct conjugation of ligands or polymers to siRNA establishes a single-component delivery system with a predefined composition. Sometimes, surface ligands can be incorporated onto nanoparticles to enhance selective targeting to diseased cells [98]. For instance, GalNAc-siRNA conjugates generated without using cationic materials are specifically internalized into hepatocytes via ligand binding.

Inorganic nanoparticles consisting of calcium phosphate, gold, carbon, and iron oxides transport siRNAs effectively due to their small size and high permeability compared to the those of liposomes and polyplexes. Effective delivery systems can be designed on the basis of the elements, bonds, and functional groups necessary. The effectiveness, stability, and precise sequence complementarity of siRNA can be enhanced by synthesis with modified nucleotides. The components of short-stranded siRNA systems also include hydrophobic modification marks and tertiary amines, and these systems show the ability to interact with short siRNA strands through various binding interactions.

Future improvements to antiviral siRNA delivery efficacy may involve (1) the use of new targeting ligands and chemical probes that specifically bind to surface markers on infected cell populations, (2) increasing the efficacy of siRNA uptake into the cytoplasm, (3) developing materials with low toxicity to widen the antiviral therapeutic window, (4) designing materials with defined degradation products that can be metabolized for repeated dosing, (5) simplifying the formulation procedure, and (6) exploring delivery to organs other than the liver [99–101].

### Side effect challenges

Many issues must be considered when siRNAs are designed, including sequence space conservation, the siRNA sequence, and siRNA and target mRNA structure constraints [102]. Combining the targeting properties of delivery vehicles with the specificity of RNAi strategies may lead to the design of products with better therapeutic efficacy and fewer side effects [103]. Additionally, chemical modifications can eliminate the immunogenicity of antiviral siRNA and increase serum stability and cell permeability [35]. ElHefnawi et al. suggested an in silico design and selection protocol complemented by an automated scoring algorithm for optimizing RNAi efficacy and reducing potential side effects [104].

Despite extensive in silico efforts, unintended siRNA activity is often observed in vitro and in vivo, and extensive preclinical testing of siRNAs in a variety of in vitro, ex vivo, and in vivo models with complex genomes is critical [72]. Unfortunately, despite extensive testing, occasional unintended side effects cannot be avoided.

For example, unintended suppression of nontarget genes, also known as "off-target effects," can cause difficulties in data interpretation and may induce toxicity. In addition to the specificity and efficiency of the silencing potency of siRNAs, pharmacodynamic-related problems include off-target RNAi activity and immunotoxicity triggered by antiviral siRNA carriers [105]. It is important to consider biological events to prevent off-target effects induced by siRNAs [106].

#### Off-target effects

The side effects of RNAi-based drugs can result from multiple factors, including off-target effects [107–110]. They can lead to unintended gene silencing and deleterious effects, raising many safety concerns. Sequence selection is a major tactic to enhance the effectiveness of antiviral siRNA and reduce off-target effects. siRNAs potentially target unintended genes that share homology with the target viral gene, leading to off-target effects. Antiviral siRNA introduction may also result in off-target effects by interfering with the expression of other mRNAs that show partial homology with the target mRNA. Silencing an unknown number of unintended genes is also possible [111], as the RISC shows the potential to suppress the expression of any mRNA with perfect complementary base-pair used to guide the siRNA to the strand seed region (bases 2–8 from the 5′ end) [112, 113]. Therefore, nontarget genes are inadvertently downregulated by antiviral siRNA, leading to problems in data interpretation and potential toxicity. Chen et al. demonstrated that off-target effects of RNAi correlate with the mismatch rate between dsRNA and untargeted mRNA [114]. Synthetic (exogenous) siRNAs can alter the expression profiles of several untargeted transcripts [115, 116]. For instance, Scacheri and colleagues observed significant changes in the protein levels of p53 and p21 that were unrelated to silencing the target gene [117].

##### Ensuring homology and specificity to prevent off-target effects

During the design of siRNAs, homology and specificity analyses should be performed to prevent off-target effects [103] because siRNAs, while primarily designed to target specific mRNA sequences, can inadvertently impact other transcripts showing partial complementarity with the siRNA duplex, leading to unintended off-target silencing [62]. Although siRNAs are intended to degrade mRNAs with high sequence complementarity, they might incidentally target mRNAs with similar sequences, causing unintended modulation of host gene expression [107, 117]. Jackson et al. emphasized that using siRNA sequences that partially match other transcripts can unintentionally silence those transcripts in addition to the targeted gene, leading to ambiguous results and potential harm [60]. In addition, Elmén et al. demonstrated that a locked nucleic acid, a synthetic high-affinity RNA-like analog, was compatible with the intracellular siRNA machinery and mitigated undesired, sequence-related off-target effects [75]. Moreover, this group demonstrated that locked nucleic acid-modified siRNAs targeting the SARS-CoV-1 genome exhibited higher efficiency than unmodified siRNAs.

Because the antisense strand in siRNA guides the RISC, precise sequence complementarity of this strand is pivotal for on-target RNAi and minimized off-target effects [118]. This underscores the importance of using siRNAs with high specificity and thoughtfully designed sequences to curtail off-target impacts [119]. Employing cutting-edge technological tools has significantly increased siRNA recognition by the RISC and reduced off-target effects. To rule out the possibility of off-target silencing, any clear matches between target and whole-genome sequences in the host cell should be carefully analyzed [113].

Off-target analysis should lead to the exclusion of siRNAs with potential binding affinity for human mRNAs. Recent work by Fakhr et al. provides comprehensive insights into sequence selection using specific protocols [120] or computational techniques [121] for enhancing gene silencing. Off-target RNAi activity can be minimized by designing siRNAs using bioinformatic tools, such as the Basic Local Alignment Search Tool (BLAST), to identify and remove homologous sequences in unintended mRNAs to which the 19 nt siRNA seed region can bind [122]. A BLAST search for cross-reactive 21-bp siRNA sequences can ensure siRNA target specificity [113].

##### Chemical modifications to mitigate the off-target effects of antiviral siRNAs

A particular position-specific chemical modification of siRNA, 2′-*O*-methyl ribose substitution at position 2 in the guide strand, was developed to reverse the silencing of partially complementary transcripts [60, 123] or reduce silencing of most off-target transcripts with complementarity to the siRNA guide strand [124, 125]. For instance, the addition of 2′-*O*-methyl modifications to the seed region, which consists of nucleotides 2–8 on the antisense strand, has been shown to provide protection against endonuclease activity and prevent off-target effects [60]. Song et al. also showed that the addition of the 2′-methoxyethyl group at a cleavage site increased both the specificity and silencing activity of siRNAs by facilitating specific RISC loading of the modified strand [126], showing that the combined modifications eliminated off-target effects. In addition, Iribe et al. demonstrated that 2′-*O*-methyl chemical and locked nucleic acid modifications of nucleotides in the seed region (positions 2–8) of the siRNA guide strand significantly reduced seed-matched off-target effects [127]. Yoshiaki et al. proposed a potent strategy to avert off-target effects without compromising RNAi activity by incorporating DNA or 2*'*-*O*-methyl modifications into the siRNA seed region. These two types of chemical modifications act through distinct molecular mechanisms to suppress off-target effects [128]. Their research revealed that the thermodynamic stability of nucleotides 2–5 showed the highest positive correlation with off-target effects, and nucleotides 8–14 showed the most negative correlation, revealing that the siRNA off-target effect is determined by base-pairing stabilities of two different subregions with opposite effects [129].

In recent findings, Shiohama et al. proposed that modifying the sense strand with X or Z moieties eradicated off-target effects caused by complementarity of the sense strand without impeding siRNA silencing efficiency [130]. Varley et al. showed that siRNAs incorporating azobenzene and propargyl modifications within the central region of the passenger strand notably increased strand selection. Furthermore, folic acid-modified siRNAs exhibited the best strand selection when positioned at the 3′ terminus. This study emphasized the utility of a streamlined method for assessing how novel chemical modifications impact strand-specific gene silencing by siRNAs [131].

#### Immunogenicity

Ideally, siRNAs would show absolutely specificity, thereby regulating only the target gene of interest. However, an increasing body of evidence suggests that this is not necessarily the case [110, 132]. Immune stimulation triggered by siRNAs is one of the major challenges in the development of safe RNAi-based therapeutics [62, 133]. Humans have developed several innate defense mechanisms against siRNAs in response to certain viral infections. The mammalian innate immune system can be activated by different types of nucleic acids, including siRNA duplexes. Although the immunomodulatory effects of nucleic acids may be therapeutically beneficial in some cases, excessive cytokine release and associated inflammatory syndromes can lead to undesirable side effects [134].

##### Immune responses induced by siRNA

Synthetic siRNA duplexes have been shown to have immunological effects that can induce high levels of inflammatory cytokine and type I interferon production [135]. These responses have been particularly notable following systemic administration [134].

The activation of the innate immune system by siRNAs is mediated through diverse mechanisms involving recognition by both cytoplasmic and endosomal receptors, including TLRs [136, 137]. Torre et al. observed TLR3 activation induced by the siRNA employed in their study. This activation led to the production of β-interferon and the initiation of caspase activity [138]. Pirher and colleagues demonstrated that siRNA activation was effectively blocked by antibodies targeting TLR3, indicating that siRNA bound TLR3 ectodomain binding sites, triggering receptor dimerization [139]. In contrast, direct cellular introduction of siRNA can mitigate the likelihood of an innate immune interferon response. This strategy circumvents the engagement of Dicer-induced mechanisms [78] and abrogates protein expression when long pieces (> 30 nucleotides) of dsRNA interact with intracellular RNA receptors [140].

##### siRNA-induced aberrant and nonspecific immune responses

The difficulties associated with siRNAs triggering immune responses, leading to inflammation and toxicity, remain a significant obstacles to their application as gene expression regulators. Recent findings indicate that siRNAs can also influence the translation of ectopically transcribed genes through partial complementarity, causing nonspecific interferon responses [116]. siRNAs have been reported to induce aberrant innate immunity either by the siRNA itself or by the delivery vehicle that facilitates cellular uptake of the siRNA [132, 141–143]. The immunostimulatory effects observed were sequence dependent, since only certain sequences were prone to induce inflammatory responses, while others were not [144]. Canonical siRNA duplexes have been identified as potent inducers of the innate immune system [143]. TLR-mediated recognition of siRNAs can trigger innate immune stimulation. Molecular recognition of RNA by TLR7 or TLR8 is poorly understood; however, it has been shown that certain sequence motifs found in either the passenger or guide strands of siRNA duplexes activate these TLRs [145]. This activation may result in the production of type I interferons and inflammatory cytokines, leading to systemic inflammation in vivo. The challenge of RNA-induced immunostimulation may be reduced by careful siRNA design involving modifications to the RNA backbone, selection of the proper siRNA target sequence, and refinement of delivery formulations and methods.

##### Chemical modifications that prevent siRNA-induced aberrant immune response

Unmodified or improperly formulated siRNA may activate TLR3 and adversely affect the blood and lymphatic systems [146]. Nonmodified siRNAs may activate the immune system through the TLR7 pathway in a nonspecific manner [109, 147]. Modifications can be added to the structure of an siRNA to prevent immune responses. Diverse chemical alterations to the siRNA backbone have also been studied because these modification may reduce immune activation without affecting their gene-silencing effects.

To mitigate the effects of immunostimulatory siRNA sequences, ribose modifications are commonly employed, as they effectively reduce cytokine production [145]. For example, in attempts to abolish aberrant immune reactivity, nonimmunostimulatory siRNAs that carry a 2′-*O*-ribose methylation modification have been developed [62].

The incorporation of 2′-*O*-methyl modifications into both sense and antisense strands diminishes RNase recognition [147, 148]. This modification is well tolerated across the entire siRNA duplex [82], effectively neutralizing any unintended immunostimulatory effect [148, 149]. Substituting uridine residues with a 2′-fluoro or 2′-deoxy group also decreases the immunostimulatory potential of siRNAs [150, 151].

##### siRNA dosage and purity considerations to mitigate unintended immune responses

Delivering synthetic siRNA at high doses has been shown to induce cytotoxic interferon and inflammatory cytokine secretion in a sequence-binding-dependent manner [109]. Moreover, the substantial ATP consumption can impose metabolic stress on host cells when siRNAs are constitutively expressed [152].

Since the RNAi pathway is a catalytic pathway and a single siRNA molecule can therefore bind to and regulate multiple mRNA copies, the administered dose can be reduced to reduce off‐target activity-associated toxicity [23]. Furthermore, the use of highly pure RNA therapeutics reduces the odds of inducing unwanted immune reactions. To eliminate impurities, high-performance liquid chromatography purification is utilized as it mediates the removal of dsRNA contaminants, which in turn decreases the production of type 1 interferon and proinflammatory cytokines [153].

### Antiviral resistance

An important consideration in developing antiviral RNAi therapies is the tendency of viral genomic sequences to undergo mutations that enable them to evade host immune responses. This creates problems in developing effective antivirals and vaccines, especially against RNA viruses such as HIV-1 and hepatitis C virus, which exhibit high mutation rates. In the early stages of RNAi therapy development, a single siRNA sequence was commonly used to achieve effective gene silencing. However, recent advancements have addressed viral mutations by employing multiple siRNAs. Combining siRNAs to simultaneously target several viruses and/or host targets mitigates the potential loss of effectiveness resulting from mutations at a specific target site.

#### Mutation and emergence of viruses resistant to siRNAs

There are two major obstacles in addition those mentioned above that must be overcome for siRNAs to become effective antiviral agents: (i) the short duration of antiviral activity due to the emergence of escape mutants resistant to siRNA, as long-term silencing of viral protein expression by siRNAs has been reported to result in the emergence of viruses resistant to RNAi [154, 155] and (ii) difficulty in designing a specific siRNA that is simultaneously effective for multiple viruses caused by the considerable variability of genomes between viral strains. Designing effective siRNAs that target viral sequences is a challenge for RNA viruses such as HIV-1 due to their remarkably high genetic variability.

The selection of siRNA-resistant viruses is a major concern in the use of RNAi as antiviral therapeutics. RNAi-resistant viral mutants emerge rapidly, mainly in targeted viral sequences. Previous studies describing resistant escape mutants to siRNA treatment suggest that single point mutations can diminish or even abolish the RNAi effect [155–157].

##### Designing siRNAs based on conserved viral RNA sequences

As mentioned above, target sequences that are highly conserved among virus isolates should be selected. One of the practical strategies to address both the problems of sustained silencing and the emergence of escape mutants is to design siRNA based on viral RNA sequences that are conserved and invariant across various strains [158, 159]. However, the design of efficient siRNAs to combat rapidly evolving viruses is complicated due to their substantial sequence diversity. To overcome this challenge, it is beneficial to design siRNAs that target highly conserved regions among various isolates, as these sequences are more likely to contain structural or functional elements that are essential for viral survival.

Further systematic investigations are required to identify the appropriate conserved target sites for siRNAs, thereby ensuring their universal and lasting antiviral effects. Notably, von Eije et al. observed that viral escape was profoundly restricted by the selection of highly conserved targets for their therapeutic strategy [160].

##### Consideration of accessibility of targeted RNA by siRNA

Westerhout EM et al. demonstrated that viral mutations induce alternative folding of the RNA structure that can occlude a target sequence and prevent siRNA binding, resulting in a reduction in RNAi efficiency [161]. Because the efficacy of siRNAs is influenced by secondary structure in the target transcript, targets within the structured viral genome with high accessibility should be considered [162–164]. That is, targeting conserved structural motifs with accessible regions in viral RNA may provide better inhibitory outcomes [13]. These two selection criteria force investigators to accept a suboptimal design of the siRNA molecule in some cases [154].

It has been suggested that RNAi efficiency is affected by the accessibility of the target RNA, which may be influenced by protein binding and the formation of RNA secondary structure [163–167]. RNA secondary structure, which is difficult to predict accurately across long RNA sequences, may affect antiviral siRNA efficacy. For example, secondary or tertiary structures of target mRNA, such as hairpin structures, can impact siRNA efficiency [168, 169]. Ge et al. demonstrated that different target sequences might contain structural motifs that may hinder the RNAi efficacy of siRNA, even though they are conserved gene sequences in viral proteins [13]. Another study using siRNAs targeting different structural regions of the M2 mRNA of influenza virus demonstrated different degrees of matrix RNA reduction, confirming the different efficacies of different target sequences in conserved regions [170].

##### Combinations of multiple siRNAs targeting distinct regions

Konishi et al. hypothesized that viral resistance to antiviral siRNAs may be due to low siRNA delivery efficiency or inaccessibility of the target genome by the siRNA molecule, suggesting that multitarget siRNA molecules may be more effective by lowering the likelihood that simultaneous mutations at multiple sites affect RNAi efficacy [171].

Jackson and colleagues suggested that using multiple siRNA duplexes to silence the target gene increased the likelihood of observing the desired phenotype and expression pattern [60]. This approach was validated by Xing et al., who demonstrated synergistic inhibitory effects on viral replication through the use of dual siRNAs directed against distinct regions of hepatitis C virus genes, achieving superior outcomes at lower doses compared to those achieved with single siRNA treatment [172]. In contrast, Haasnoot et al. cautioned that excessive influx of multiple siRNAs may lead to increased off-target effects and toxicity by saturating RNAi pathways [173]. In summary, it is crucial to determine the minimal effective concentration of antiviral siRNAs for gene silencing while preventing unintended effects.

#### Viral intrinsic suppressors of RNA silencing

Most insect or plant RNA viruses have evolved RNA silencing suppressors to counteract antiviral RNAi effects. Among plant viruses, the tombusvirus-encoded P19 protein blocks RNAi machinery by binding siRNAs via their dsRNA-binding domain, thereby sequestering siRNAs and preventing their action in RNAi pathways and preventing siRNA incorporation into the RISC [174, 175]. Similarly, the NS3 protein of rice hoja blanca virus and the 2b protein of tomato aspermy virus block RNAi action by binding to long dsRNA or siRNA [176, 177]. The turnip crinkle virus P38 protein has been shown to specifically block the activity of the Dicer-like 4 protein [178, 179], and cauliflower mosaic virus P6 protein disrupted the activity of RNAi machinery components [180]. Among proteins in insect viruses, the B2 protein of flock house virus blocks RNAi by dsRNA binding [181, 182], and the B2 protein of Wuhan nodavirus was identified as an RNA silencing suppressor that targets both dsRNAs and Dicer-2 [183, 184].

Although the majority of RNA silencing suppressors have been identified in plant and invertebrate viruses, an increasing number of mammalian viruses have also been found to encode suppressors. RNA silencing suppressor proteins include the nucleocapsid protein of SARS-CoV-1 [185] and SARS-CoV-2 [186], influenza A virus NS1, vaccinia virus E3L [187], the hepatitis C virus core [188, 189], Ebola virus VP35, VP35, and VP40 [190, 191]; NS4B of all four Dengue virus serotypes [192]; 3A of human enterovirus 71 [193] and HIV-1 Tat [194]; and adenovirus virus-associated RNAs I and II [195].

Cui et al. provided evidence showing that the nucleocapsid protein of SARS-CoV efficiently inhibited Dicer-mediated dsRNA cleavage and post-Dicer activity by sequestering dsRNAs and siRNAs [185]. The authors hypothesized that the coronavirus nucleocapsid protein, as an RNA silencing suppressor, might protect viral RNA from RNAi-mediated gene silencing in three stages: (i) binding viral single-stranded RNAs to prevent unnecessary intramolecular and intermolecular dsRNA conversion into positive- and negative-sense genomic or subgenomic RNAs; (ii) shielding virus-derived dsRNA from Dicer cleavage through suppressor dsRNA binding activity; and (iii) binding virus-derived siRNAs to interfere with RISC assembly.

Furthermore, Haasnoot et al. showed that adenovirus virus-associated RNAs inhibit RNAi by acting as decoy substrates for Exportin 5, Dicer, and the RISC [195, 196]. Ebola virus VP35 and HIV-1 Tat are thought to block Dicer activity [190, 194], whereas influenza A virus NS1 and vaccinia virus E3L may sequester dsRNAs and siRNAs [187, 197]. The capsid protein of Venezuelan equine encephalitis virus contains regions that bind specifically to RNA, and this protein leads to an increased in the sequestration of siRNA and therefore increased resistance to RNAi [198]. Samuel et al. demonstrated that the capsid protein of medically important flaviviruses, including yellow fever virus, Zika virus, and West Nile virus, inhibited RNA silencing by interfering with Dicer [199]. Qiu et al. identified nonstructural protein 3A of human enterovirus 71 as an RNA silencing suppressor that inhibited Dicer-mediated siRNA biogenesis by sequestrating dsRNAs [193].

Although viral RNA silencing suppressors may counter RNAi effects in the context of natural infections, these factors may not necessarily diminish the effectiveness of siRNA-based antiviral therapeutics [190]. Interestingly, Nishitsuji et al. showed that HIV-1 Tat only suppressed shRNA-elicited RNAi treatment by functionally inhibiting host Dicer activity but not siRNA-elicited RNAi effects [194, 200]. Similarly, Lu et al. presented evidence demonstrating that RNA silencing suppressors potently inhibited RNAi effects induced by shRNAs or human miRNA precursors but did not affect RNAi effects induced by artificial (synthetic) siRNAs [189, 195]. Chen et al. also suggested that the use of presynthesized siRNAs might be more efficient than the use of shRNAs [189]. These findings suggest that the RNAi approach should be carefully considered as an antiviral therapeutic strategy and that the use of presynthesized siRNAs may be more efficient than the shRNA-based approach. Most RNA-silencing suppressor proteins identified from mammalian viruses possess interferon- or protein kinase R-antagonistic properties, and they are essential for replication and pathogenesis [201–204]. Haasnoot et al. noted that high concentrations of exogenous synthetic siRNAs, for example, to block Ebola virus replication, will saturate VP35 RNA silencing suppressors, rendering VP35 ineffective and subsequently inhibiting virus replication [190, 205].

## Conclusion

The use of siRNA to silence genes has emerged as a powerful approach for studying cellular processes and targeting disease-causing factors with precision. Over the past twenty years, there have been notable breakthroughs in the development of siRNA-based therapeutics for a range of diseases, including viral infection. RNAi is a gene silencing mechanism that provides a powerful means to specifically inhibit viral infection. Increasing evidence has emerged to suggest that RNAi pathways are evolutionarily conserved defense mechanisms against pathogenic viral infections [206], providing a tremendous opportunity for the development of oligonucleotide-based drugs. However, siRNA-based therapeutic approaches are limited by several challenges, including limited stability, inefficient cellular uptake, off-target effects, the potential for stimulating the immune system, and the possibility of the emergence of escape mutant viruses [207–209].

To enhance the cellular uptake of siRNAs, synthetic nanoparticles made up of polymers, lipids, and conjugates can be employed, along with the integration of cell-specific targeting ligands in carriers [78]. For example, chemical modifications, such as 2′-fluoro and thioate linkages, can be utilized to prolong the half-life of siRNAs and increase their stability [210]. Identification of the cellular pathways of RNA immunorecognition may facilitate the development of strategies to prevent the inclusion of immunostimulatory oligonucleotide motifs during siRNA design [135]. For long-term inhibition, multiple siRNA expression vectors can be utilized [211]. Bioinformatics approaches can be employed to identify potential target sites and design siRNAs with optimal features for initial experiments [212].

To use siRNA as an antiviral therapy, side effect concerns, including off-target effects, must be addressed. Although it is possible to design virus-specific siRNAs that do not cross-react with the human genome, this possibility needs to be confirmed. There is a risk of side effects if an siRNA partially hybridizes with untargeted mRNAs, so a balance between potency and safety must be considered when formulating the final product. In vivo studies are necessary to investigate any potential inflammatory responses to antiviral siRNA treatment. Given the constantly evolving viral genome, it is important to target specific and highly effective therapeutics at conserved genomic regions to inhibit viral replication [213]. In addition, combinations of multiple siRNAs targeting separate regions of the genome can alleviate the problem of resistant mutants, and the use of a therapeutic cocktail increases the likelihood of activity retention against newly emergent viral strains.

Selectively silencing genes by hijacking the endogenous RNAi pathway with exogenous antiviral siRNAs has become a widely used strategy to study gene function, and further, this approach has shown impressive therapeutic potential that is expected to be realized soon. The biotechnology industry has devoted significant resources to the development of siRNA therapeutics for treating various diseases, including viral infections, and an increasing number of studies have focused on the development of antiviral siRNAs in recent years. Despite obstacles to the use of siRNA antiviral therapy, it is anticipated that the future of RNAi therapeutics is bright because of interdisciplinary efforts and technological advancements. Therefore, it can be projected that antiviral siRNA applications will provide enormous potential for the treatment of viral infections in the future.

## Data Availability

All data relevant to this review are included in the text, references, table, and figures.

## Abbreviations

ATP: Adenosine triphosphate
BLAST: Basic Local Alignment Search Tool
COVID‐19: Coronavirus disease 2019
DNA: Deoxyribonucleic acid
dsRNA: Double-stranded RNA
GalNAc: *N*-Acetylgalactosamine
GC: Guanine‐cytosine
HIV: Human immunodeficiency virus
kDa: Kilodaltons (molecular weight)
MERS-CoV: Middle East respiratory syndrome coronavirus
miRNA: MicroRNA
mRNA: Messenger RNA
nt: Nucleotide
RISC: RNA-induced silencing complex
RNA: Ribonucleic acid
RNAi: RNA interference
SARS-CoV: Severe acute respiratory syndrome coronavirus
SARS-CoV-2: Severe acute respiratory syndrome coronavirus 2
shRNA: Short hairpin RNA
siRNA: Small interfering RNA
TLR: Toll-like receptor
